# Meta-analysis of plant-derived exosome-like nanoparticles for the treatment of ulcerative colitis: efficacy and mechanisms insights

**DOI:** 10.3389/fphar.2026.1845981

**Published:** 2026-06-22

**Authors:** Xuefei Hu, Jian Wang, Mao Guo, Chuang Guan, Yangyang Wang, Junran Wang, Yu Wu, Shuwen Han

**Affiliations:** 1 Department of Basic Medical Sciences, Tarim University, Aral, China; 2 Faculty of Pharmacy, Xinjiang Second Medical College, Karamay, China; 3 Huzhou Central Hospital, Affiliated Central Hospital Huzhou University, Huzhou, China

**Keywords:** inflammatory factors, meta-analysis, plant-derived exosome-like nanoparticles, systematic review, ulcerative colitis

## Abstract

**Background:**

Plant-Derived Exosome-Like nanoparticles (PELNs) are vital bioactive vesicles from medicinal plants, containing protective flavonoids, polyphenols, alkaloids, terpenoids, saponins, polysaccharides, and microRNAs. These compounds play crucial roles in PELNs’ anti-inflammatory and other pharmacological effects. PELNs show promise as a treatment in animal experiments for ulcerative colitis (UC) due to their biocompatibility, low immunogenicity, and natural targeting abilities. This review examines their therapeutic effects on UC and the potential mechanisms behind their action.

**Methods:**

This study synthesises existing data through a meta-analysis, providing comprehensive preclinical evidence for the use of PELNs in ulcerative colitis. To identify studies examining the effects of PELNs on UC, the researchers conducted a comprehensive search of five databases.

**Results:**

This search identified a total of 25 eligible studies involving 304 animal subjects, with data up to 15 September 2025. According to the results of the meta-analysis, PELNs demonstrated significant effects in mitigating weight loss, increasing colon length, and reducing faecal consistency scores. Further mechanistic studies suggest that PELNs may exert their effects through multiple pathways. In addition to influencing inflammatory factors and the mucosal barrier, PELNs may also modulate the microbiota during the repair process of UC. PELNs intervention resulted in a statistically significant change in the Chao index (*p* = 0.05) and a highly significant change in the Shannon index (*p* < 0.0001).

**Conclusion:**

This meta-analysis suggests that, in animal models of ulcerative colitis (UC), PELNs may be associated with antioxidant, anti-inflammatory, immunomodulatory and intestinal barrier-protective effects. This study only supports the correlational results derived from preclinical animal studies, and cannot confirm the definite action mechanisms nor clinical efficacy in humans. Further basic researches are still needed for in-depth exploration. Visit https://inplasy.com/inplasy-2025-11-0075/ to access the systematic review registration, labeled as INPLASY2025110075.

**Systematic Review Registration:**

Identifier INPLASY2025110075.

## 1 Introduction

Ulcerative colitis (UC) is a global disease (Wangchuk et al., 2024; Li et al., 2025a). In recent years, with a marked increase in global incidence rates, patients’ ability to work and quality of life have been severely compromised (Bhala et al., 2022; Weidner et al., 2024). Disruption of the intestinal barrier function, immune imbalance, and dysbiosis of the gut microbiota may all contribute to the development of UC (Ma et al., 2023). The existing standard treatment options include anti-inflammatory agents, immunosuppressive therapies, and biologic agents that act against tumor necrosis factor (TNF). Although these therapies have demonstrated efficacy in symptom relief and disease control, their effectiveness is often inconsistent and accompanied by considerable adverse effects and high treatment costs (Zheng et al., 2024). Aminosalicylates, for instance, might lead to gastrointestinal issues such as abdominal pain and diarrhea (Abe et al., 2023); glucocorticoids are associated with complications including diabetes and glaucoma (Rutgeerts, 2001); immunosuppressants carry risks of hepatotoxicity (Fournier et al., 2010). Consequently, the investigation and development of novel therapeutic strategies targeting UC remain of paramount clinical importance.

PELNs are nanoscale with sizes of approximately 30–150 nm in diameter. There are lipid bilayer vesicles extracted from plants, which closely resemble exosomes (Han et al., 2025; Liu et al., 2025). These vesicles encapsulate a variety of bioactive molecules with significant biological functions. Proteins, nucleic acids, lipids, and small molecules are included, and PELNs are involved in various pathological processes as well as intercellular communication. Recent research indicates that PELNs show therapeutic effects in animal models of UC (Zu et al., 2021; Zhang et al., 2016; Zhu et al., 2023; Zhu et al., 2025). Owing to their excellent biocompatibility, low immunogenicity and inherent targeting properties, PELNs may serve as a potential delivery system and therapeutic intervention for UC (Zhao et al., 2025); their characteristics have also attracted attention in the fields of biology and medicine (Yi et al., 2023).

This research compiles current data on the therapeutic effects of PELNs in animal models. Furthermore, it summarizes and analyzes the potential mechanisms of action, thereby providing scientific, and reliable preclinical evidence for the application of PELNs in UC.

## 2 Methods

The manuscript adheres to PRISMA guidelines for systematic reviews and meta-analyses and is registered with INPLASY. The number is INPLASY2025110075. The website is https://inplasy.com/inplasy-2025-11- 0075/.

### 2.1 Literature search

In accordance with the PRISMA (Preferred Reporting Items for Systematic Reviews and Meta-Analyses) guidelines, we conducted a search of five databases—PubMed, Web of Science, Embase, the Cochrane Library and ScienceDirect—to collect detailed data on preclinical animal studies investigating the use of PELNs for the treatment of UC. The search was conducted up to 15 September 2025. Inclusion criteria were as follows: studies must have been published in English. Two authors independently screened the titles and abstracts of relevant studies, followed by a screening of the full texts. In the event of disagreement, a third author acted as a referee. A specialist librarian was consulted to ensure that the search syntax included all synonyms related to PELNs and UC. The search strategy is detailed in the supplementary materials.

### 2.2 Inclusion criteria

(1) Study Population: Animal models of UC established in mice and rats using common methods such as DSS and TNBS; experimental animals must be free from underlying comorbidities such as diabetes, immunodeficiency or gene knockout. (2) Study Design: A published randomised controlled animal trial. (3) Intervention Method: The intervention group received PELNs; there were no restrictions on dosage, route of administration or duration of intervention. (4) Control Group Setup: A model blank control or solvent control group was established, with clear group differentiation. (5) Data Requirements: The outcome measure data in the literature must be complete, with valid values available for extraction for use in meta-analysis.

### 2.3 Exclusion criteria

(1) Human clinical trials, reviews, case reports and conference abstracts. (2) Studies adopting non-plant-derived exosomes or exosome-like nanoparticles as interventions. (3) Studies without animal models, blank/positive control groups or reasonable experimental design. (4) Studies without accessible full texts or incomplete key outcome data.

### 2.4 Data extraction

The information was independently extracted by two authors. The data including as the following: (1) First author and year of publication; (2) Species, gender, and number of animals; (3) Method used for modeling UC in animals; (4) Intervention strategies, such as the cycle and dosage of intervention; (5) Evaluations of outcome measures encompassed assessments of disease activity index, body weight, and colon length. As well as other inflammation-related markers, the expression of intestinal barrier proteins and alterations in the gut microbiota. The chart data were quantified using GraphPad Prism 10 software. The basic information of the included literature is shown in Table 1.

**TABLE 1:** Basic characteristics of the included studies.

Study (year)	Plant-derived	Species	Model method	Outcome index
Zhu et al. (2025)	Andrographis paniculata	C57BL/6 mice	3% DSS in drinking water (7 days)	①②③④⑥⑦⑧⑪ ⑯
Zu et al. (2021)	tea	FVB female mice	3% DSS in drinking water (7 days)	①②③④⑦⑧⑨⑪ ⑫ ⑭
Zhu et al. (2023)	Portulaca oleracea	C57BL/6 male mice	3% DSS in drinking water (7 days)	①②③④⑤⑥⑦⑧⑪ ⑯
Zhang et al. (2016)	Ginger	FVB/NJ female mice	1.5% DSS in the drinking water (7 days)	①⑤⑥⑦⑧⑨
Zhang et al. (2024)	Sophora flavescens	C57BL/6 mice	3% DSS in drinking water (7 days)	①②③⑥⑦⑧
Weng et al. (2025)	American ginseng	Balb/c mice	3% DSS in drinking water (7 days)	①②③⑨⑪
Wei et al. (2025)	Polygonatum sibiricum	Male C57BL/6J mice	2.5% DSS in drinking water (8 days)	①②③⑨⑰ ⑱ ⑲
Wang et al. (2024)	Garlic	Male C57BL/6J mice	3% DSS in drinking water (37 days)	①②③⑦⑧
Tuo et al. (2025)	Tangerine peel	Male C57BL/6J mice	2.5% DSS in drinking water (7 days)	①③⑨⑰ ⑱
Tang et al. (2025)	Rhubarb	Female C57BL/6 mice	3% DSS in drinking water (5 days)	①②③⑥⑦⑧⑨⑪ ⑭ ⑮
Sik Kim et al. (2024)	Boehmeria japonica	Female C57BL/6 mice	2.5% DSS in drinking water (7 days)	①②③
Peng et al. (2025)	Paederia scandens	Male Balb/c mice	3% DSS in drinking water (7 days)	①⑤⑥⑦⑧
Mao et al. (2021)	Ginger	C57BL/6 mice	2% DSS in drinking water (16 days)	①②③⑧
Lu et al. (2024)	Edible pueraria lobata	Male C57BL/6J mice	3% DSS in drinking water (5 days)	①②③
Lu et al. (2025)	Turmeric	Mice	3% DSS in drinking water (7 days)	①②③⑥⑦⑧
Li Y. et al. (2025)	Folium Artemisiae Argyi	Female Balb/c mice	2.5% DSS in drinking water (12days)	①②③⑥⑦⑧⑪ ⑰ ⑱
Li J. et al. (2024)	Houttuynia cordata	Male C57BL/6 mice	3% DSS in drinking water (7 days)	①②③④⑤⑦⑧⑭ ⑮
Kim et al. (2023)	Ginseng	Balb/C male mice	2.5% DSS in drinking water (7days)	①②③⑰ ⑱
Kang et al. (2024)	Allium tuberosum	C57BL/6 female mice	3% DSS in drinking water (7 days)	①②③⑤⑥⑦⑧⑨⑰ ⑱
Huang et al. (2024)	Ginger	Male C57BL/6 mice	2.5% DSS in drinking water (7days)	①②③⑤⑥⑦⑧⑨⑪ ⑱
Gong et al. (2025)	Zanthoxylum bungeanum	Male C57BL/6J mice	2.0% DSS in drinking water (7days)	①②③④⑥⑦⑧
Gao et al. (2022)	Turmeric	ICR mice	3.5% DSS in drinking water (12days)	①②③⑤⑥⑦⑧⑪ ⑰ ⑱
Eom et al. (2022)	Hemp	Male C57BL/6 mice	5% DSS in drinking water (7days)	①⑮
Choi et al. (2023)	Aloe	Male C57BL/6 mice	5% DSS in drinking water (7days)	①②③⑧
Zhu et al. (2023)	Garlic	Male C57BL/6J mice	3.0% DSS in drinking water (7days)	①②③⑥⑦⑧⑨⑩⑭ ⑮

① CL; ② DAI; ③ BWC; ④ stool consistency score; ⑤ IL-10; ⑥ IL-1β; ⑦ IL-6; ⑧ TNF-α; ⑨ spleen weight; ⑩ IL-17; ⑪ MPO; ⑫ MDA; ⑬ SOD; ⑭ ZO-1; ⑮ occludin; ⑯ IL-12; ⑰ chao; ⑱ shannon; ⑲ simpson.

### 2.5 Quality assessment

The quality of the included animal studies was assessed utilizing a 10-item risk of bias (RoB) tool developed by the Systematic Review Center for Laboratory Animal Experimentation (SYRCLE). This tool evaluates the following criteria: (A) sequence generation; (B) baseline characteristics; (C) allocation concealment; (D) random housing; (E) blinding of investigators; (F) random outcome assessment; (G) blinding of outcome assessors; (H) incomplete outcome data; (I) selective outcome reporting; and (J) other sources of bias. Each criterion is assigned a score of one point (Hooijmans et al., 2014).

### 2.6 Data analysis

This meta-analysis was conducted using Comprehensive Meta-Analysis (CMA) and Review Manager 5.4 software. The primary outcome measures from animal studies—body weight change (BWC), colon length (CL), disease activity index (DAI) and stool consistency scores—were all continuous variables; the standardized mean difference (SMD) and 95% confidence interval (CI) were used to assess the effect of PELNs on UC. Other outcome measures were also assessed as continuous variables. A significance threshold of 0.05 was set when comparing the PELNs intervention group with the control group. Heterogeneity was assessed using the *I* ^*2*^ statistic. When *I* ^*2*^ ≤ 50%, a fixed-effects model was used for analysis; when *I* ^*2*^ > 50%, a random-effects model was used; when *I* ^*2*^ > 75%, subgroup analysis and sensitivity analysis were conducted to identify the sources of heterogeneity. Publication bias was assessed using funnel plots and the Egger test. If study heterogeneity was high, subgroup analysis was first conducted based on basic study characteristics to preliminarily screen for sources of heterogeneity; if heterogeneity within subgroups persists after stratification, meta-linear regression analysis is further employed to quantify the extent to which various confounding factors account for the heterogeneity; finally, sensitivity analysis is conducted using the stepwise exclusion method to test the robustness and reliability of the pooled effect size results.

## 3 Results

### 3.1 Literature investigation and selection

**FIGURE 1:**
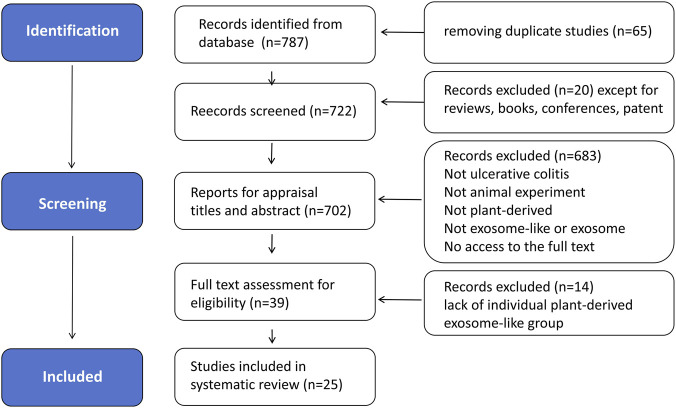
The flowchart of the literature search and screening process.

The search strategy identified 787 English-language articles: 190 from PubMed, 160 from Embase, 420 from Web of Science, four from the Cochrane Library, and 13 from other sources. After removing duplicates, 722 articles were kept in EndNote. Following the evaluation of titles, abstracts, and full texts, 702 articles were discarded. After thoroughly evaluating the full texts, 25 articles were finally selected. The process of searching and screening the literature is shown in Figure 1.

### 3.2 Features of the included studies

This study included 304 animals from 25 researches. Those animals were equally divided into the intervention group and the model group. The collection of animals was made up of 230 C57BL/6 or C57 BL/6J mice, 12 FVB mice, 10 FVB/NJ mice, 36 Balb/c mice, and 16 ICR mice. Regarding sex specificity, 13 studies used only male animals, seven studies focused solely on female animals, and five studies did not specify the sex of the animals. The dosage range of PELNs administered ranged from 0.1 to 1,000 mg/kg. Oral gavage was used in twenty-one studies, rectal administration was used in two studies, and two studies did not mention the administration route. Interventions lasted between 5 and 37 days, with six studies conducting long-term interventions of at least 14 days, while 19 studies involved short-term interventions of fewer than 14 days.

Throughout the study, PELNs treatment was administered to the experimental group, and the model group experienced solvent-induced UC. The UC model was consistently developed by incorporating dextran sulfate sodium (DSS) into the drinking water. Twenty-five studies evaluated CL, while 21 focused on BWC: 21 studies examined DAI, and five looked at stool consistency. In the analysis of inflammatory biomarkers, IL-10 levels were reported in eight studies, IL-1β in 13 studies, IL-6 in 16 studies, TNF-α in 18 studies, and MPO activity in eight studies. Five studies analyzed ZO-1 and occludin levels as indicators of the mucosal barrier. Additionally, the Chao index was evaluated in six studies, and the Shannon index was assessed in seven studies. Table 1 offers comprehensive details about the studies included (Zhu et al., 2025; Zu et al., 2021; Zhu et al., 2023; Zhang et al., 2016; Zhang et al., 2024; Weng et al., 2025; Wei et al., 2025; Wang et al., 2024; Tuo et al., 2025; Tang et al., 2025; Sik Kim et al., 2024; Peng et al., 2025; Mao et al., 2021; Lu et al., 2024; Lu et al., 2025; Li et al., 2025b; Li et al., 2024a; Kim et al., 2023; Kang et al., 2024; Huang et al., 2024; Gong et al., 2025; Gao et al., 2022; Eom et al., 2022; Choi et al., 2023; Zhenzhu et al., 2023).

### 3.3 Study quality

Study quality was independently evaluated by two authors using the 10-item risk of bias (RoB) tool developed by the Systematic Review Center for Laboratory Animal Experimentation (SYRCLE). Out of the 25 studies reviewed, 17 referenced randomization of animals without specifying the method used, seven studies omitted details on animal grouping, and one study grouped animals by weight, introducing a substantial risk of bias. Table 2 offers a comprehensive overview of the methodological quality of the included studies.

**TABLE 2:** Methodological quality of included animal studies (SYRCLE tool).

Study (year)	A	B	C	D	E	F	G	H	I	J	Scores
Zhu et al. (2025)	1	1	0	1	0	0	0	1	1	1	6
Zu et al. (2021)	1	1	0	1	0	0	0	1	1	1	6
Zhu et al. (2023)	1	1	0	1	0	0	0	1	1	1	6
Zhang et al. (2016)	1	1	0	1	0	0	0	1	1	1	6
Zhang et al. (2024)	0	1	0	1	0	0	0	1	1	1	5
Weng et al. (2025)	1	1	0	1	0	0	0	1	1	1	6
Wei et al. (2025)	1	1	0	1	0	0	0	1	1	1	6
Wang et al. (2024)	1	1	0	1	0	0	0	1	1	1	6
Tuo et al. (2025)	1	1	0	1	0	0	0	1	1	1	6
Tang et al. (2025)	1	1	0	1	0	0	0	1	1	1	6
Sik Kim et al. (2024)	0	1	0	1	0	0	0	1	1	1	6
Peng et al. (2025)	1	1	0	1	0	0	0	1	1	1	6
Mao et al. (2021)	0	1	0	1	0	0	0	1	0	1	4
Lu et al. (2024)	1	1	0	1	0	0	1	1	1	1	7
Lu et al. (2025)	0	1	0	1	0	0	0	1	1	1	5
Li Y. et al. (2025)	1	1	0	1	0	0	0	1	1	1	6
Li J. et al. (2024)	1	1	0	1	0	0	0	1	1	1	6
Kim et al. (2023)	1	1	0	1	0	0	0	1	1	1	6
Kang et al. (2024)	1	1	0	1	0	0	0	1	1	1	6
Huang et al. (2024)	1	1	0	1	0	0	0	1	1	1	6
Gong et al. (2025)	1	1	0	1	0	0	0	1	1	1	6
Gao et al. (2022)	0	1	0	1	0	0	0	1	1	1	6
Eom et al. (2022)	1	1	0	1	0	0	0	1	1	1	6
Choi et al. (2023)	1	1	0	1	0	0	0	1	1	1	6
Zhu et al. (2023)	1	1	0	1	0	0	0	1	1	1	6

The methodological quality of included studies. A, sequence generation; B, baseline characteristics; C, allocation concealment; D, random housing; E, blinding of experimentalists; F, random outcome assessment; G, blinding of outcome assessors; H, incomplete outcome data; I, selective outcome reporting; J, other sources of bias.

### 3.4 Outcomes of the meta-analysis

#### 3.4.1 Primary outcome indicator

**FIGURE 2:**
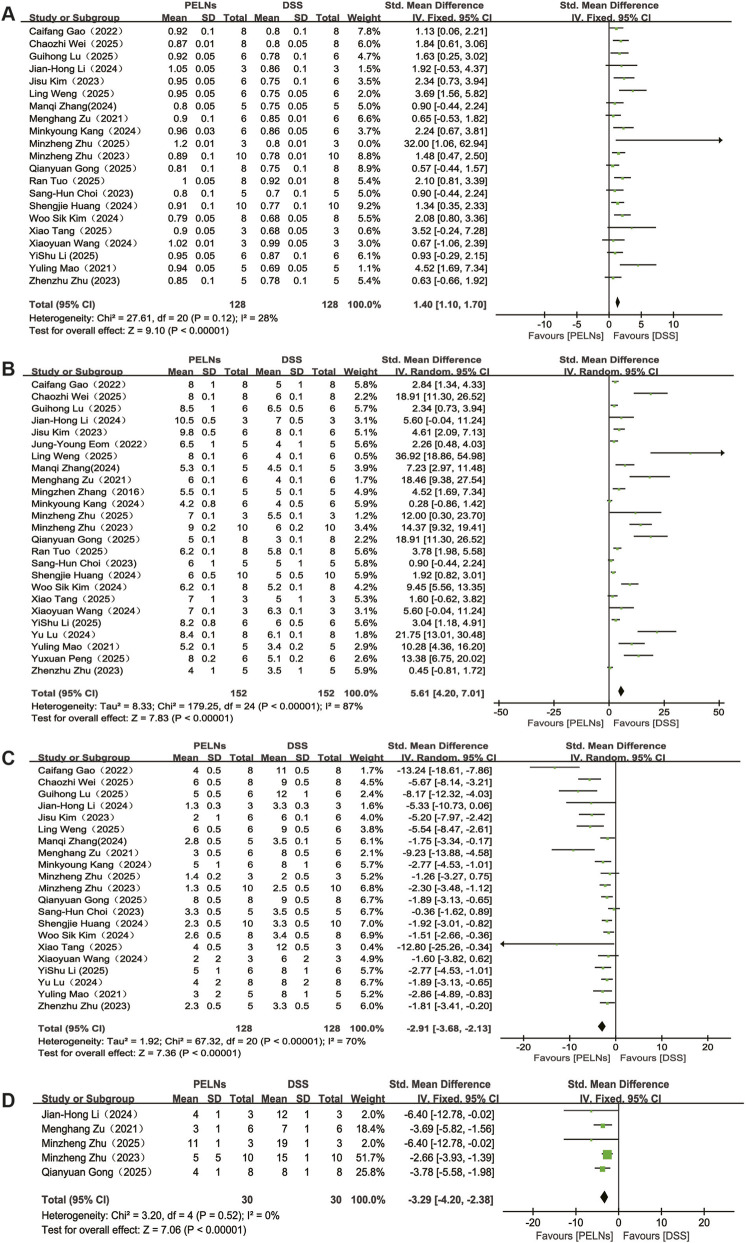
Forest plots showing results of histological and primary outcomes. **(A)** Body weight change (BWC). **(B)** Colon length (CL). **(C)** Disease activity index (DAI). **(D)** stool consistency scores. The squares and horizontal lines represent the standardized mean difference (SMD) and 95% confidenceintervals (CIs); the diamond denotes the pooled effect.

In UC animal models, BWC, CL, DAI, and stool consistency scores serve as critical indicators of UC severity. These indicators allow for a quick assessment of the overall health status of mice through visual observation and represent the most fundamental evaluation criteria. All the primary outcome measures are shown in Figure 2.

BWC may function as an indirect metric for assessing the impact of intestinal inflammation on the overall health status of animals with UC by reflecting their general nutritional condition. The research utilized a random-effects model to investigate how PELNs affect BWC in UC animals. In total, 21 studies were conducted to explore the influence of PELNs on BWC in UC animals, encompassing 256 animals. The meta-analysis indicated a more pronounced drop in BWC in the DSS group, whereas PELNs notably improved BWC in UC animals (SMD = 1.40, 95% CI [1.10, 1.70], *p* < 0.00001), as depicted in Figure 2A.

A total of 25 studies with 304 animals investigated the impact of PELNs on colonic length in UC animals, showing a significant reduction in CL (SMD = 5.61, 95% CI [4.20, 7.01], *p* < 0.00001) (Figure 2B).

The DAI score, a key indicator of intestinal inflammation, includes assessments of body weight changes, stool consistency, and fecal occult blood. A meta-analysis of 21 studies involving 256 animals showed that the PELNs intervention significantly reduced the DAI score in UC animals, indicating its effectiveness in alleviating intestinal inflammation (SMD = −2.91, 95% CI [-3.68, −2.13], *p* < 0.00001) (Figure 2C).

The meta-analysis of fecal consistency scores, involving 60 animals, is shown in Figure 2D. Results showed significantly lower stool consistency scores in the PELNs group compared to the DSS group, indicating stool characteristics closer to normal in the PELNs group (SMD = −3.29, 95% CI [-4.20, −2.38], *p* < 0.00001) (Figure 2D). The PELNs group showed significantly greater efficacy than the DSS group, with statistically significant differences.

#### 3.4.2 Inflammation-related indicators

**FIGURE 3:**
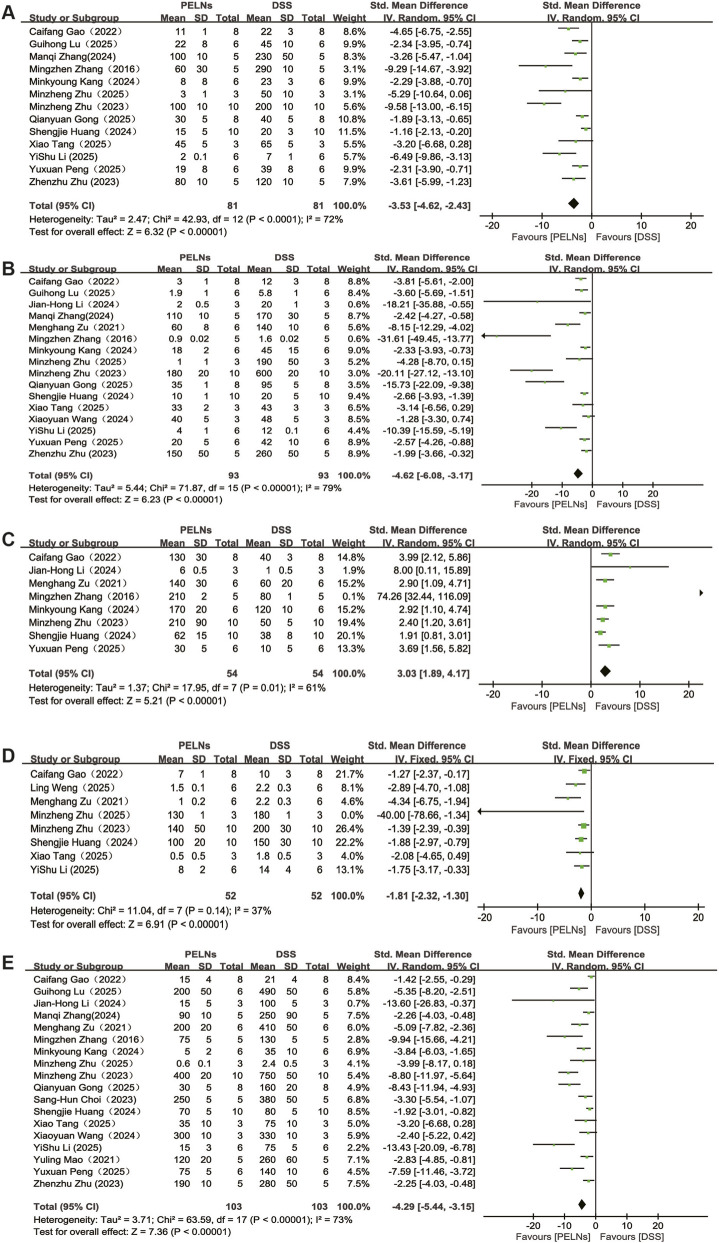
Forest plots showing results of Inflammation-related indicators. **(A)** IL-1β. **(B)** IL-6. **(C)** IL-10. **(D)** MPO. **(E)** TNF-α.

IL-1β (Figure 3A), IL-6 (Figure 3B), IL-10 (Figure 3C), TNF-α (Figure 3E) and MPO (Figure 3D) serve as crucial inflammatory biomarkers. IL-1β, primarily secreted by immune cells, is a crucial pro-inflammatory cytokine involved in inflammatory responses and immune regulation. IL-6 plays a role in both promoting and reducing inflammation, being involved in immune responses, inflammation, and tissue repair. IL-10 is an anti-inflammatory cytokine that suppresses pro-inflammatory factor release, maintains immune balance, and mitigates inflammatory damage. TNF-α is a potent pro-inflammatory cytokine involved in initiating inflammation and modulating the immune response; however, elevated levels may result in inflammatory storms. MPO is primarily present in neutrophils, participating in the body’s anti-infection and inflammatory responses, and serves as an inflammatory marker. Forest plots of inflammatory biomarker results are shown in Figure 3.

#### 3.4.3 Intestinal barrier-related parameters

**FIGURE 4:**
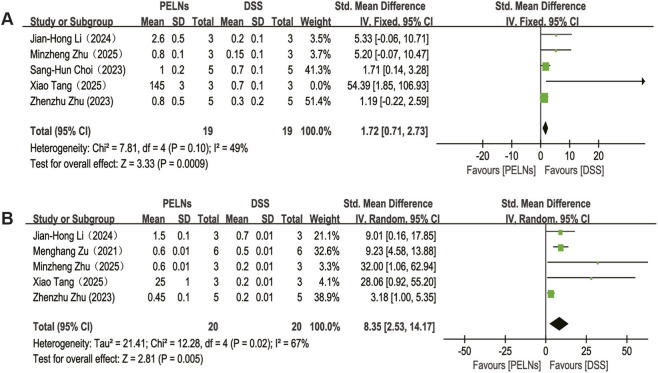
Forest plots showing results of intestinal barrier-related parameters. **(A)** Occludin. **(B)** ZO-1.

ZO-1 and Occludin are essential tight junction proteins in colonic epithelial cells, crucial for preserving intestinal barrier integrity by controlling selective substance passage. PELNs intervention resulted in a significant enhancement of Occludin protein expression in the UC animal model, according to the results (n = 38, SMD = 1.72, 95% CI [0.71, 2.73], *I* ^*2*^ = 49%, *p* < 0.00001). The data suggests a possible rise in ZO-1 levels (n = 40, SMD = 8.35, 95% CI [2.53, 14.27], *I* ^*2*^ = 67%, *p* < 0.00001) (Figure 4). This indicates that PELNs might aid in restoring the compromised intestinal epithelial barrier function in UC.

#### 3.4.4 Gut microbiota-related parameters

**FIGURE 5:**
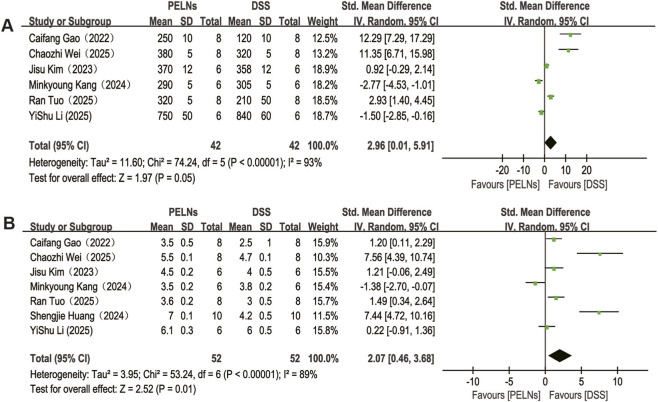
Forest plots showing gut microbiota-related parameters. **(A)** Chao index. **(B)** Shannon index.

The Chao and Shannon indices are widely utilized metrics for evaluating gut microbiota diversity. The Chao index is typically expressed as an alpha diversity index, reflecting “how many species” are present in a community. The Shannon index is a composite diversity measure representing both species richness and evenness, with higher values indicating greater community diversity. This study used a meta-analysis to investigate the effects of PELNs on gut microbial diversity in a model of ulcerative colitis (UC). Figure 5 indicates that the meta-analysis revealed PELNs significantly improve both the Chao index (n = 84, SMD = 2.96, 95% CI [0.01, 5.91], *I* ^*2*^ = 93%, *p* = 0.05) and the Shannon index (n = 104, SMD = 2.07, 95% CI [0.46, 3.68], *I* ^*2*^ = 89%, *p* < 0.0001).

### 3.5 Publication bias

**FIGURE 6:**
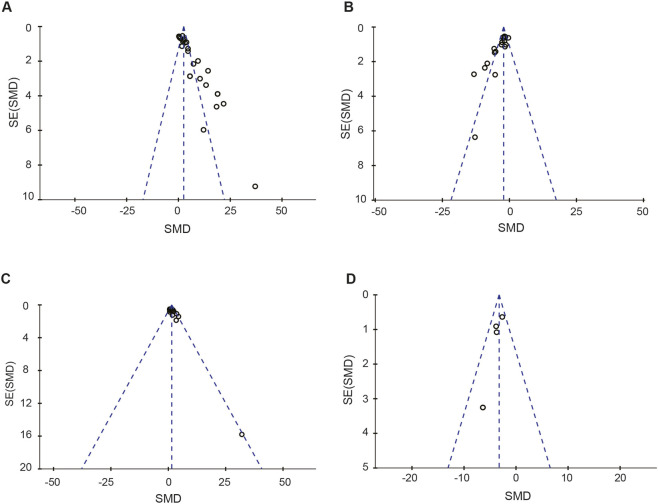
The funnel plot of primary outcome measures. **(A)** Colon length (CL). **(B)** Disease activity index (DAI). **(C)** Body weight change (BWC). **(D)** stool consistency score.

Funnel plots, along with Egger’s test, were utilized to uncover possible publication bias in the primary outcome measures. The effect sizes for CL and DAI fell outside the boundaries of the funnel plot, indicating the presence of asymmetry. In contrast, the effect sizes for both BWC and stool consistency score were entirely contained within the funnel plot limits, suggesting symmetry. Figure 6 shows potential publication bias for CL and DAI (*p* < 0.001), but none for BWC or stool consistency score. The Classic fail-safe N test shows minimal publication bias impact on the CL parameter, indicating robust conclusions. Both the Begg and Mazumdar rank correlation test and Egger’s regression test reveal significant publication bias in the CL outcomes. This implies that studies with small sample sizes, null effects, or negative effects may have been excluded from the analysis, thereby introducing bias into the current meta-analysis findings. After applying the trim-and-fill method to address bias, the estimated effect size became more conservative, necessitating a reassessment of the study’s conclusions based on these adjusted findings.

The Begg and Mazumdar rank correlation test indicated a significant publication bias in DAI. A significant negative correlation existed between effect size precision and magnitude, suggesting that many low-precision positive studies may have been published, while high-precision negative studies might have been excluded from analysis. The trim and fill method by Duval and Tweedie revealed no missing studies requiring adjustment in this meta-analysis, indicating that the overall effect remained statistically significant after accounting for publication bias.

### 3.6 Sources of heterogeneity in the literature

For results showing a high degree of heterogeneity (CL and DAI), we investigated the sources of this high heterogeneity in the primary outcome measures through subgroup analysis, meta-regression and sensitivity analysis.

#### 3.6.1 Subgroup analysis, regression analysis and sensitivity analysis of colon length (CL)

The 25 included studies exhibited high overall heterogeneity (I^2^ = 87%), and a random-effects model was used for the meta-analysis. (1) Subgroup analysis revealed that (see Table 3) following stratification by animal strain, drug concentration, duration of intervention and modelling method, heterogeneity remained high in most subgroups (*I* ^*2*^ > 85% in all cases), with the exception of the subgroup modelled with DSS concentrations >3%, where heterogeneity was reduced (*I* ^*2*^ = 54.87%). This suggests that the aforementioned factors are not the primary sources of heterogeneity. (2) Further univariate meta-regression analysis showed that animal strain, drug concentration, duration of intervention, and modelling method showed no significant association with heterogeneity for the CL outcome (all *p* > 0.05), failing to explain the observed high heterogeneity. (3) Sensitivity analysis results showed that the pooled effect size and level of heterogeneity did not change significantly after sequentially excluding individual studies. In summary, the results of this meta-analysis on CL outcomes are relatively stable. 1ts findings hold some value as a reference.

**TABLE 3:** Subgroup analysis of the standardized mean difference (SMD) for colon length (CL) and disease activity index (DAI).

Outcome	Subgroup	Category	No. of studies	SMD [95% CI]	*p*	I^**2**^
CL	Species	Balb/c	4	4.71 [ 3.37, 6.06]	<0.0001	90.54%
		C57BL	17	2.73 [ 2.24, 3.23]	<0.0001	90.88%
		Others	4	2.22 [1.34, 3.10]	<0.0001	90.50%
	Concentration (mg/kg)	≥50	8	3.16 [2.44, 3.89]	<0.0001	90.54%
		<50	15	2.49 [1.98, 3.04]	<0.0001	90.52%
	Treatment days	<14	18	7.92 [5.91, 9.93]	<0.0001	89.73%
		≥14	7	4.49 [2.19, 6.78]	<0.0001	90.38%
	Model method	0.025	6	5.81 [3.28, 8.34]	<0.0001	88.47%
		0.03	13	8.81 [5.99, 11.63 ]	<0.0001	92.29
		>3%	3	2.12 [0.86, 3.37]	0.0010	54.87%
		≤2%	3	11.60 [3, 40, 19.80 ]	0.0060	89.03%
DAI	Species	C57BL	15	−2.33 [-2.98, -1.68]	<0.0001	60.65%
		Others	6	−7.40 [-10.24, -4.56]	<0.0001	82.09%
	Concentration (mg/kg)	<50	13	−4.29 [−5.70, −2.88]	<0.0001	85.07%
		≥50	6	−2.10 [-2.68, −1.53]	<0.0001	0%
		No mention	2	−5.80 [-7.63, −3.97]	<0.0001	0%
	Treatment days	<14	15	−4.41 [-5.74, −3.09]	<0.0001	85.06%
		≥14	6	−2.24 [-2.82, −1.65]	<0.0001	0%
	Model method	2.50%	5	−3.38 [−4.84, -1.82]	<0.0001	78.27%
		3.00%	12	−3.67 [−4.86, -2.47]	<0.0001	75.75%
		>3%	2	−6.98 [-20.30, 6.34 ]	0.3040	96.33%
		≤2%	2	−2.35 [−3.40, −1.30]	<0.0001	5.61%

#### 3.6.2 Subgroup analysis, meta-regression and sensitivity analysis of the disease activity Index (DAI)

The 21 included studies exhibited an overall heterogeneity of 70%, and a random-effects model was used to pool the effect sizes. Subgroup analysis results showed (see Table 3): (1) Species subgroup: the SMD for the C57BL/6 mice was −2.33 (95% CI [−2.98, −1.68], *p* < 0.0001, *I* ^*2*^ = 60.65%), and the SMD for the “others” was −7.40 (95% CI [−10.24, −4.56], *p* < 0.0001, *I* ^*2*^ = 82.09%), these results suggest that animal species may contribute to the observed heterogeneity. (2) Concentration subgroup: in the <50 mg/kg subgroup, the SMD was −4.29 (95% CI [−5.70, −2.88], *p* < 0.0001, *I* ^*2*^ = 85.07%), with high heterogeneity; in the ≥50 mg/kg subgroup, heterogeneity disappeared (*I* ^*2*^ = 0%), and the SMD was −2.10 (95% CI [−2.68, −1.53], *p* < 0.0001), suggesting that high-dose administration may be a significant source of heterogeneity. (3) Treatment duration subgroup: treatment duration <14 days, the SMD was −4.41 (95% CI [−5.74,−3.09], *p* < 0.0001, *I* ^*2*^ = 85.06%), with high heterogeneity; for the subgroup with intervention duration ≥14 days, heterogeneity disappeared (*I* ^*2*^ = 0%), with an SMD of −2.24 (95% CI [−2.82, −1.65], *p* < 0.0001), suggesting that treatment duration is one of the key sources of heterogeneity. (4) Model method subgroup: heterogeneity was significantly reduced in the DSS concentration ≤2% subgroup (*I* ^*2*^ = 5.61%), with an SMD of −2.35 (95% CI [−3.40, −1.30], *p* < 0.0001); whereas *I* ^*2*^ remained as high as 75.75%–96.33% in the subgroups with DSS concentrations of 2.50%, 3.00% and >3%, suggesting that modelling with low-concentration DSS can significantly reduce heterogeneity.

Despite substantial between-study heterogeneity, sensitivity and publication bias analyses suggested that the results were not strongly influenced by outliers or potential publication bias. The pooled effect size reached statistical significance, indicating that the intervention may have a beneficial effect in animal models of colitis, though these findings should be interpreted cautiously due to the high heterogeneity and preclinical nature of the included studies.

**FIGURE 7:**
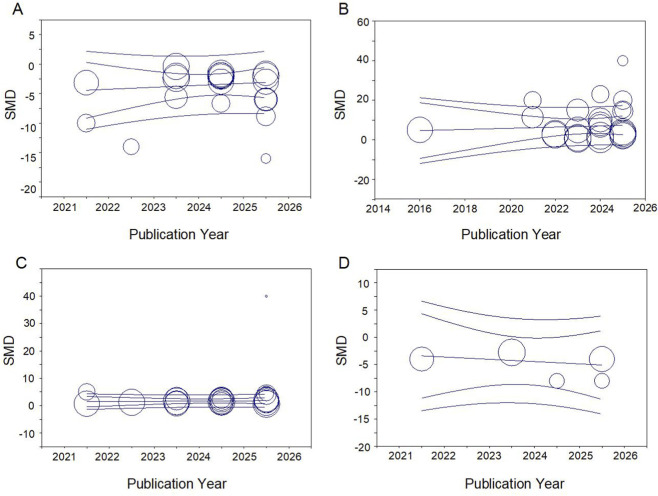
Univariate meta-regression of the publication year of major outcome indicators. **(A)** Disease activity index (DAI). **(B)** Colon length (CL). **(C)** Body weight change (BWC). **(D)** stool consistency score.

Through univariate meta-regression, we evaluated whether the publication year influenced heterogeneity and found no effect on the heterogeneity of primary outcomes (Figure 7).

## 4 Discussion

### 4.1 Summary of evidence

We conducted a systematic review of 25 studies to evaluate the effectiveness of initial treatments and the potential mechanisms of PELNs in managing UC. UC is a chronic, recurrent, and refractory disease. Initial treatment should aim to decrease diarrhea frequency, enhance body weight, manage bloody stools, support mucosal healing, and relieve clinical symptoms (Li et al., 2024b).

Data support that PELNs may enhance CL (SMD = 5.61, 95% CI [4.20, 7.01], *p* < 0.00001), increase BWC (SMD = 1.40, 95% CI [1.10, 1.70], *p* < 0.00001), and reduce the DAI score (SMD = −2.91, 95% CI [-3.68, −2.13], *p* < 0.00001). The results also indicate that PELNs appear to have a positive effect on faecal consistency scores (SMD = −3.29, 95% CI [−4.20, −2.38], p < 0.00001), suggesting a potential role in alleviating systemic clinical symptoms in a model of ulcerative colitis. This effect may be mediated through the regulation of multiple pathways associated with intestinal inflammation.

### 4.2 Potential mechanisms of action

#### 4.2.1 Suppressing abnormal intestinal inflammation and regulating immune imbalance

PELNs can reduced levels of IL-1β (SMD = −3.53, 95% CI [-4.62, −2.43]), IL-6 (SMD = −3.08, 95% CI [-3.65, −2.51]), MPO (SMD = −1.81, 95% CI [-2.32, −1.30]), TNF-α (SMD = −4.29, 95% CI [-5.44, −3.15]), and increased IL-10 (SMD = 3.03, 95% CI [1.89, 4.17]), all with *p* < 0.00001. These changes suggest that PELNs may exert a potential regulatory effect on UC by inhibiting inflammatory cell infiltration, promoting mucin secretion, and modulating relevant signalling pathways (Ma et al., 2023; Wang et al., 2024; Feng et al., 2025). However, there is considerable heterogeneity in the pooled effect sizes of various inflammatory markers; therefore, this trend should be viewed with caution, and the relevant research findings should be interpreted with due care.

#### 4.2.2 Promoting damaged epithelial regeneration and repairing the intestinal mucosal barrier

The results of this meta-analysis indicate a upward trend in the expression levels of Occludin (SMD = 1.72, 95% CI [0.71, 2.73], *I* ^*2*^ = 49%, *p* < 0.00001); the tight junction protein ZO-1 also exhibited upregulation (SMD = 8.35, 95% CI [2.53, 14.27], *I* ^*2*^ = 67%, *p* < 0.00001). These statistical differences were derived from data based on 38 and 40 experimental animals, respectively. Based on the available pooled data, PELNs may, to some extent, upregulate the expression of the two tight junction proteins, Occludin and ZO-1, in intestinal epithelial tissue. These changes in expression may help to improve the condition of damaged intestinal epithelium, stabilise the structure of the intestinal mucosal barrier, and thereby mitigate damage caused by intestinal inflammatory stimuli, potentially playing a positive role in the restoration of intestinal mucosal barrier function (Gao et al., 2025). However, due to the large effect sizes of some indicators and the presence of moderate heterogeneity, this regulatory effect requires further validation through additional research.

#### 4.2.3 Modulating gut microbiota to increase SCFA content

Current research suggests that PELNs may exert a potential therapeutic effect on UC by modulating the structure of the gut microbiota and increasing the abundance of beneficial bacteria (Zhu et al., 2023). The Chao-1 index and the Shannon index are commonly used measures of gut microbial diversity; the former reflects species richness, whilst the latter reflects overall diversity and the evenness of distribution within the microbiota. Both indices are generally low in UC, but show some recovery following PELNs intervention, suggesting that the number of gut microbial species and community homeostasis have improved to some extent (Wang et al., 2024; Li et al., 2023).

The results of this meta-analysis show that following PELNs intervention, both the Chao-1 index (SMD = 2.96, 95% CI [ 0.01, 5.91], *I* ^*2*^ = 93%, *p* = 0.05) and the Shannon index (SMD = 2.07, 95% CI [ 0.46, 3.68], *I* ^*2*^ = 89%, *p* < 0.0001) both showed an upward trend following PELNs intervention, suggesting that microbial richness and diversity are crucial factors in maintaining the balance of the gut microbiome (Li et al., 2023). It should be noted that the pooled analysis of these two indices exhibited extremely high heterogeneity; therefore, the observed trends should be treated as indicative only and should not be over-interpreted. Based on this, it is speculated that the role of PELNs in regulating the gut microbiota may be associated with increased levels of short-chain fatty acids such as acetic acid, propionic acid and butyric acid (Zhu et al., 2025).

### 4.3 Analysis of sources of heterogeneity

This study conducted subgroup analyses for colon length (CL) and the Disease Activity Index (DAI) due to their high levels of heterogeneity. The results of the subgroup analyses revealed significant differences between studies in terms of animal model design, intervention protocols and outcome measures.

Regarding the CL outcome, although heterogeneity remained high within each subgroup following subgroup analysis by animal strain, drug concentration and duration of intervention, this indicates that these factors are not the primary sources of overall heterogeneity. Subgroup analysis of modelling methods, however, revealed a significant reduction in heterogeneity (*I* ^*2*^ = 54.87%) within the high-concentration DSS (>3%) subgroup, suggesting that variations in DSS concentration may be a key factor influencing heterogeneity in CL outcomes. Different concentrations of DSS can lead to varying degrees of colonic inflammation and damage, thereby affecting the extent of changes in colon length; this is a critical aspect requiring standardisation in future research.

With regard to DAI outcomes, the results of the subgroup analysis indicate that concentration, duration of treatment and modelling methods may all influence heterogeneity. It is worth noting that heterogeneity for both concentration (≥50 mg/kg) and duration of treatment (≥14 days) fell to 0%, suggesting that differences in intervention protocols are the primary source of heterogeneity in DAI outcomes; whilst heterogeneity in the low-concentration DSS (≤2%) model also decreased significantly (*I* ^*2*^ = 5.61%), indicating that standardised modelling methods are crucial for reducing heterogeneity in DAI outcomes. Furthermore, heterogeneity among other mouse models (*I* ^*2*^ = 82.09%) was significantly higher than that in the C57BL/6 mouse model (*I* ^*2*^ = 60.65%), suggesting that differences in animal models may also exert some influence on heterogeneity.

In summary, the subgroup analysis in this study suggests that modelling methods, concentrations and duration of intervention are key factors influencing heterogeneity in this meta-analysis. Future animal studies should, as far as possible, adopt standardised modelling methods, intervention protocols and outcome measurement criteria to reduce heterogeneity between studies and improve the reliability and comparability of results.

### 4.4 Limitations of the study and potential sources of bias

The pooled results for most outcome measures in the meta-analysis exhibited a high degree of heterogeneity between studies; neither subgroup analyses based on pre-specified factors nor meta-regression analyses were able to effectively explain the primary sources of this heterogeneity. It is speculated that the heterogeneity primarily stems from numerous unaccounted-for differences in the details of the included studies, including variations in animal modelling procedures, the baseline physiological status of experimental animals, daily housing conditions, methods of outcome measurement, and inconsistencies in the preparation, isolation and purification processes of PELNs across studies, among other potential confounding factors. Despite the marked heterogeneity among the included studies, this study employed a random-effects model for the meta-analysis of effect sizes, and the aggregated results still hold some academic value.

Furthermore, this study has several limitations and potential sources of bias. The standardized mean difference (SMD) obtained for some outcome measures are relatively large, and such results should be interpreted with caution. On the one hand, there is a clear small-sample effect; pilot studies with smaller sample sizes are more likely to yield overly optimistic positive results, thereby leading to an overestimation of the overall intervention effect. On the other hand, publication bias is prevalent in this field; studies with significant positive conclusions are more likely to be published, whilst those with negative or non-statistically significant results are difficult to comprehensively collect and include, further inflating the overall pooled effect size. Due to the combined influence of the aforementioned sources of bias, the conclusions drawn from this study should not be over-generalised; further validation is required through high-quality studies with standardised designs, adequate sample sizes and consistent experimental procedures.

### 4.5 Current challenges and future prospects

This meta-analysis included only preclinical animal studies; the findings merely indicate that plant-derived exosome nanoparticles exert certain effects in animal models of UC. They do not elucidate the specific mechanisms of action *in vivo*, nor do they directly confirm their efficacy and safety in humans. Given the significant differences between animals and humans at the physiological and metabolic levels, and the heterogeneity in experimental design and administration methods across the included studies, the conclusions of this study cannot be directly extrapolated to clinical practice. Further in-depth mechanistic studies and high-quality basic validation trials are required to further explore their potential for clinical translation.

PELNs may offer a new avenue of research for the treatment of UC (Zhu et al., 2025). These nanovesicles are characterised by low immunogenicity, tolerance to the gastrointestinal environment, and the ability to target and accumulate in intestinal tissues. Existing research suggests that PELNs may exert their effects through multiple synergistic mechanisms, including the regulation of inflammatory responses, repair of the intestinal mucosal barrier, and modulation of the gut microbiota, and are expected to provide new avenues for intervention studies related to UC.

## 5 Conclusion

**FIGURE 8:**
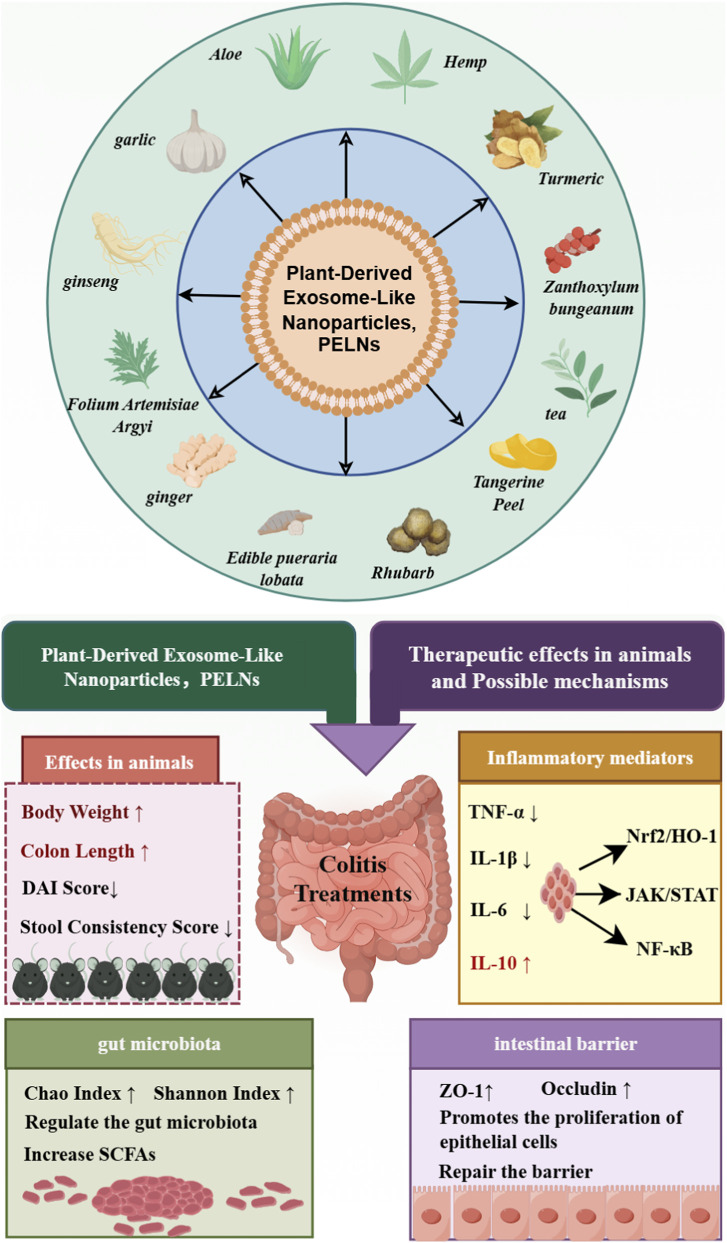
An overview of the mechanism of action of Plant-Derived Exosome-Like nanoparticles (PELNs) in UC.

To summarize, this research utilized a randomized controlled trial with animal models to thoroughly examine the therapeutic effectiveness and the potential mechanisms of PELNs in the treatment of UC. The findings suggest that PELNs may alleviate clinical symptoms in UC models via multiple mechanisms (see Figure 8), which offers potential insights for drug development and clinical intervention strategies for this disease. However, it is important to acknowledge that the current experimental design has room for improvement. Future research should rigorously adhere to the standards and guidelines governing randomized controlled trials. Since the current evidence quality and bias risk are not yet ideal, the results should be interpreted carefully. The path toward clinical translation remains challenging; nonetheless, these unresolved scientific questions offer clear directions and innovative opportunities for subsequent investigations.

## Data Availability

The original contributions presented in the study are included in the article/Supplementary Material, further inquiries can be directed to the corresponding authors.
