# Supplementary material for: Meta-analysis of plant-derived exosome-like nanoparticles for the treatment of ulcerative colitis: efficacy and mechanisms insights
Source: Front Pharmacol. 2026 Jun 22;17:1845981. doi: 10.3389/fphar.2026.1845981 (PMC13333397; doi:10.3389/fphar.2026.1845981)
Supplement: Supplementary file 1 [file Supplementaryfile1.docx]

**Supplementary Materials**

Table 1 :Literature search strategy for Plant-Derived Exosome-Like nanoparticles in Ulcerative Colitis

| **Search Strategy (PubMed)** | |
| --- | --- |
| #1 | **Colitis [Mesh]** |
| #2 | Colitis Gravis [Title/Abstract] |
| #3 | Idiopathic Proctocolitis [Title/Abstract] |
| #4 | Inflammatory Bowel Disease, Ulcerative Colitis Type [Title/Abstract] |
| #5 | Ulcerative Colitis [Title/Abstract] |
| #6 | #1or#2or#3or#4or#5 |
| #7 | **Exosome** [Mesh] |
| #8 | Condensing Vacuole [Title/Abstract] |
| #9 | Vacuole, Condensing [Title/Abstract] |
| #10 | Granule, Zymogen [Title/Abstract] |
| #11 | Microvesicle, Synaptic-Like[Title/Abstract] |
| #12 | SLMVs[Title/Abstract] |
| #13 | Granule, Secretory[Title/Abstract] |
| #14 | #7or#8or#9or#10or#11or#12or#13 |
| #20 | #6and#14 |
|  |  |

**Figure S1 Subgroup analysis of CL regarding species**

**Figure S2 Subgroup analysis of CL regarding concentration**

**Figure S3 Subgroup analysis of CL regarding treatment days**

**Figure S4 Subgroup analysis of CL regarding model method**

**Figure S5 Subgroup analysis of DAI scores regarding species**

**Figure S6 Subgroup analysis of DAI scores regarding concentration**

**Figure S7 Subgroup analysis of DAI scores regarding treatment days**

**Figure S8 Subgroup analysis of DAI scores regarding model method**
